# Comparative Survival Outcomes of Hyperthermic Intraperitoneal Chemotherapy, Intraperitoneal Chemotherapy and Intravenous Chemotherapy for Primary Advanced Ovarian Cancer: A Network Meta-Analysis

**DOI:** 10.3390/jcm12031111

**Published:** 2023-01-31

**Authors:** Qin Tang, Mao Huang, Jing Zhang, Zhen Huang, Linlian Wang, Zhengxin Gong, Liangdan Tang

**Affiliations:** Department of Obstetrics and Gynecology, The First Affiliated Hospital of Chongqing Medical University, Chongqing 400016, China

**Keywords:** ovarian cancer, hyperthermic intraperitoneal chemotherapy, intraperitoneal chemotherapy, intravenous chemotherapy, survival outcomes

## Abstract

Objective: We aimed to compare the survival outcomes and adverse events of hyperthermic intraperitoneal chemotherapy (HIPEC), intraperitoneal chemotherapy (IP)and intravenous chemotherapy (IP)for primary advanced ovarian cancer. Methods: PubMed, CENTRAL (Cochrane Central Registry of Controlled Trials), Embase, Web of Science and Scopus were searched using multiple terms for primary advanced ovarian cancer, including randomized controlled trials and comparative studies in both Chinese and English (up to date 15 August 2022). Outcomes include overall survival, progression-free survival and adverse events. The data were pooled and reported as hazard ratio (HRs) with 95% confidence intervals. The Newcastle–Ottawa Scales were used to assess the risk of bias in the included comparative study. The Cochrane Collaboration’s Risk of Bias Tool was used for randomized controlled trials. Results: In total, 32 studies, including 6347 patients and 8 different platinum-based chemotherapy regimens, were included in this network meta-analysis. Our analysis results showed that HIPEC2 (carboplatin with area under the curve 10) exhibited a statistically significant OS benefit compared to IV, weekly dose-dense chemotherapy and HIPEC1 (cisplatin with 75/100 mg/m^2^). Intraperitoneal plus intravenous chemotherapy was associated with a statistically significantly better likelihood of overall survival compared to IV. For progression-free survival, our statistical results only suggest a better progression-free survival in ovarian cancer patients treated with HIPEC1 compared with weekly dose-dense chemotherapy. No evidence of difference was observed between the other comparison groups. Compared with the non-HIPEC group, HIPEC may had a higher incidence of electrolyte disturbances (≥grade 3). Conclusion: Our statistical analysis suggests that the groups receiving HIPEC2 had a better OS than the groups receiving IV, weekly dose-dense chemotherapy and HIPEC1. For PFS, our analysis only showed HIPEC1 is better than IV. Moreover, HIPEC may lead to a higher incidence of electrolyte disturbances (≥grade 3). HIPEC therapy for advanced ovarian cancer is currently controversial.

## 1. Introduction

Ovarian cancer is the third most common gynecologic malignancy worldwide and is the most lethal one. Worldwide, there were an estimated 31.4 thousand new ovarian cancer cases and almost 20.7 thousand deaths from ovarian cancer in 2020 [1]. Most ovarian cancer cases, especially epithelial ovarian cancer (EOC), are diagnosed at an advanced stage when the tumor has spread to the peritoneal cavity and upper abdominal organs [2]. The mainstay treatment of ovarian cancer is still cytoreductive surgery (CRS) combined with platinum-based chemotherapy [3].

The metastatic spread of ovarian cancer to the peritoneum carries a poor prognosis. Hyperthermic intraperitoneal chemotherapy (HIPEC) is an intraoperative procedure that directly delivers chemotherapy in a heated solution to the abdominal cavity after CRS, which is thought to improve penetration of the peritoneal surface and improve chemotherapeutic agent absorption and susceptibility of cancer cells [4]. Combined with CRS, HIPEC improves oncologic outcomes for patients with peritoneal surface malignancy from gastric cancer [5], mesothelioma [6], and pseudomyxoma peritonei [7]. However, whether HIPEC improves outcomes for patients with advanced ovarian cancer remains controversial. In addition to the traditional platinum-based intravenous chemotherapy (IV), a variety of other chemotherapy regimens are available. For patients with stage III ovarian cancer with no residual mass greater than 1.0 cm, intravenous paclitaxel plus intraperitoneal cisplatin and paclitaxel improves patients’ survival [8]. Andrew et al. hold the idea that weekly dose-dense chemotherapy can be delivered successfully as first-line treatment for epithelial ovarian cancer [9].

Although there have been published meta-analyses comparing the efficacy and safety of HIPEC vs. IP and HIPEC vs. IV in the treatment of advanced ovarian cancer [10]. However, there is no network meta-analysis comparing HIPEC, IV, intraperitoneal chemotherapy (IP), weekly dose-dense chemotherapy and other chemotherapy regimens. Therefore, we conducted a network meta-analysis (NMA), including several platinum-based chemotherapy regimens, to directly and indirectly compare the survival outcomes for primary advanced ovarian cancer.

## 2. Materials and Methods

### 2.1. Data Sources and Search Strategy

This network meta-analysis was carried out in accordance with the extension of the preferred reporting items for systematic reviews and meta-analyses (PRISMA) statement for network meta-analyses [11].

We systematically searched PubMed, CENTRAL, Embase, Web of Science and Scopus. A systematic search using a combination of key words and terms for “Ovarian Neoplasms”, “Hyperthermic Intraperitoneal Chemotherapy”, “Intraperitoneal Chemotherapy” and “Intravenous Chemotherapy”. Studies published before 15 August 2022 were searched. Our detailed search strategies are provided in File S1. Taking PubMed as an example, the specific search strategy is shown as follows:

#1 (“Ovarian Neoplasms”[Mesh]) OR ((“Neoplasms”[Mesh] OR carcinoma*[tw] OR neoplas*[tw] OR tumour* [text word, tw] OR sarcoma*[tw] OR adenoma*[tw] OR tumor*[tw] OR cancer*[tw] OR oncolog*[tw] OR malignan*[tw] OR metasta*[tw] OR carcinogen*[tw] OR oncogen*[tw]) AND (ovrian*[tw] OR ovary[tw]))

#2 (chemotherapy[tw] OR chemoperfusion[tw]) AND (intraperitoneal*[tw] OR intra-peritoneal*[tw] OR peritoneal*[tw])

#3 HIPEC [tw]

#4 #2 OR #3

#5 #1 AND #4

### 2.2. Inclusion and Exclusion Criteria

Literature screening was performed separately by two investigators, and disagreements were settled by discussion with a third investigator. The literature was selected with the following criteria: (1) patients newly diagnosed with ovarian cancer, primary peritoneal, or fallopian tube carcinoma; (2) patients who underwent CRS (with or without neoadjuvant chemotherapy), and the residual disease of ≤1 cm in diameter; (3) patients who underwent platinum-based chemotherapy, including HIPEC, IV, IP and weekly dose-dense chemotherapy; (4) survival data and adverse events are available; (5) published English or Chinese literature was included. Meanwhile, the literature with the following criteria were excluded: (1) data were incomplete or could not be used for statistical analysis; (2) duplicate publications, reviews, abstracts, letters, and comments, etc.; (3) literature in non-English or non-Chinese languages; (4) studies with fewer than 10 patients; (5) partients included recurrent ovarian cancer.. References of the included papers were further searched to identify other potentially relevant studies.

### 2.3. Data Extraction and Quality Evaluation

Data extraction and quality assessment were performed separately by two investigators, and disagreements were resolved through discussions with a third investigator. Data were extracted using a standard excel form, including the first author’s name, year of publication, time of study, stage of ovarian cancer, number of patients, mean age, study design, follow-up time, and survival outcomes. Survival data were extracted using a hazard ratio (HR) with a 95% confidence interval (CI) from included studies. If HR and 95% CI were not reported directly, we extracted the data from the Kaplan–Meier curve using Engauge Digitizeit 4.1 and calculated HR and 95% CI as described by Tierney [12]. Unadjusted or univariate analysis results for HRs were considered for the aggregation of the survival data.

We used the Newcastle–Ottawa Scale (NOS) to evaluate the risk of bias of included comparative studies. The Cochrane Collaboration’s Risk of Bias Tool was used for randomized controlled trials (RCTs) [13].

### 2.4. Statistical Analysis

Analyses were performed using the Stata 14.0 (StataCorp, College Station, TX, USA) and R 4.1.3 software (R Foundation for Statistical Computing, Beijing, China, “meta” and “netmeata” and “gemtc” packages). The data of overall survival (OS) and progression-free survival (PFS) were pooled using hazard ratio (HRs) and corresponding 95% CI. The dichotomous data results were pooled and reported as relative risk (RRs) with 95% CIs. When there is a closed loop, the consistency test is conducted between the direct comparison and the indirect comparison. Consistency between the direct and indirect evidence was also assessed by comparing the individual data point’s posterior mean deviance contributions for the consistency and inconsistency model and the node-splitting analysis.

## 3. Results

### 3.1. Characteristics of Included Studies

**Figure 1:**
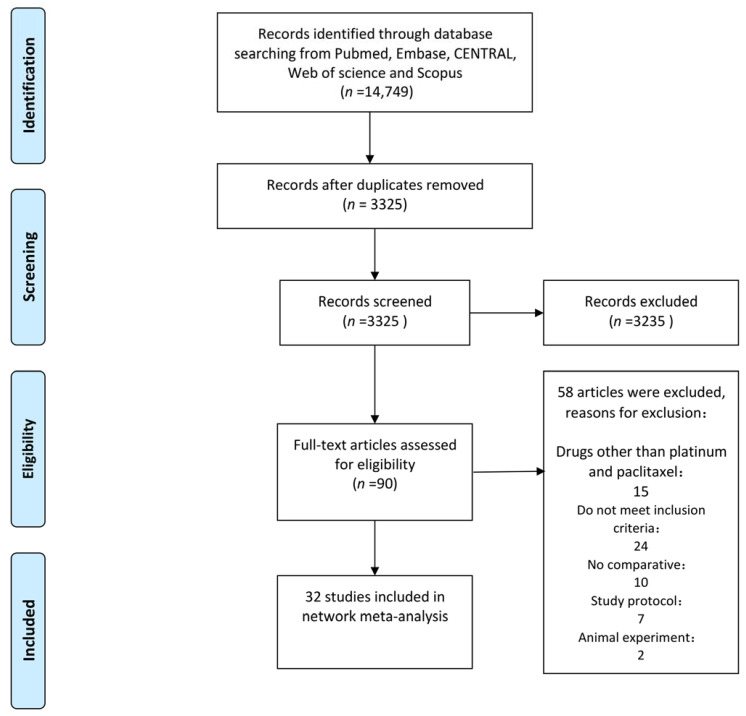
Flow diagram of study selection.

In total, 32 studies, published between 2001 and 2022, were included in the analysis, enrolling a total of 7718 women with newly diagnosed ovarian cancer. Among the included studies, there were 23 comparative studies and 9 RCTs. The study of Manning-Geist BL (2021) et al. (11) reported survival outcomes for miliary and non-miliary disease spread, respectively. Lee J (2018) et al. (12) reported survival outcomes for interval debulking surgery (IDS) and primary debulking surgery (PDS) plus IV or IP, respectively. Thus, we consider them as two studies separately. To further compare the effect of different chemotherapy regimens on survival outcomes of ovarian cancer patients, we divided them into the following groups: HIPEC1, cisplatin (75/100 mg/m^2^) was perfused with a target temperature of 41–43 °C for 90 min; HIPEC2, carboplatin area under the curve (AUC) 10 was perfused with a target temperature of 41–43 °C for 90 min; HIPEC3, cisplatin (100 mg/m^2^) and paclitaxel (175 mg/m^2^) was perfused at the temperature of 41.5 °C for 90 min; HIPEC4, paclitaxel (60 mg/m^2^) were perfused with a target temperature of 41–43 °C for 90 min; IV, intravenous paclitaxel (175 mg/m^2^) and carboplatin AUC (5 or 6) or cisplatin (75 mg/m^2^) every 21 days; IP, intraperitoneal cisplatin (100 mg/m^2^) or intraperitoneal cisplatin on day 1 plus intraperitoneal paclitaxel on day 8; IVIP, day1: intravenous paclitaxel (135 mg/m^2^) over a 3 or 24 h, day2: intraperitoneal cisplatin (75/100 mg/m^2^) or carboplatin AUC (5 or 6), day8: intraperitoneal paclitaxel (60 mg/m^2^) every 21 days; DD (weekly dose-dense chemotherapy), day1: intravenous carboplatin (AUC 5–6), day 1, 8, 15: intravenous paclitaxel 80 mg/m^2^. There were 3 three-arm studies and 29 dual-arm studies. For OS, this study includes six closed loops, which are IV-IVIP-HIPEC2, IV-IVIP-DD, IV-IVIP-DD-HIPEC2, IV-HIPEC1-HIPEC2, IVIP-DD-HIPEC2 and IV-HIPEC2-HIPEC4. For PFS, this study includes four closed loops, which are IV-IVIP-HIPEC, IVIP-DD-HIPEC2, IV-IVIP-DD-HIPEC2 and IV-IVIP-DD. The characteristics of the included studies are shown in Table 1. The study selection flowchart (PRISMA) is shown in Figure 1.

### 3.2. Network Map

**Figure 2:**
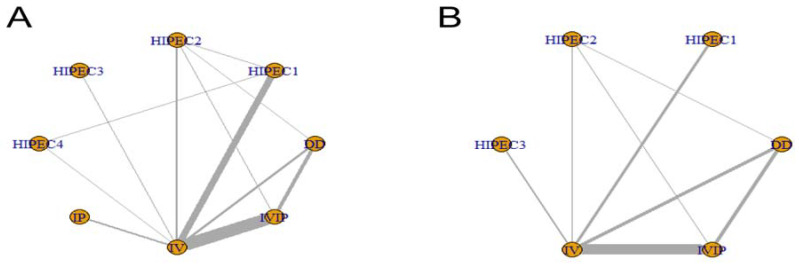
Network map of OS (**A**) and PFS (**B**).

The line between two nodes represents a direct comparison. The thicker the line, the more research. The larger the node, the larger the sample size. The network maps for OS and PFS are shown in Figure 2.

### 3.3. Overall Survival (OS)

**Figure 3:**
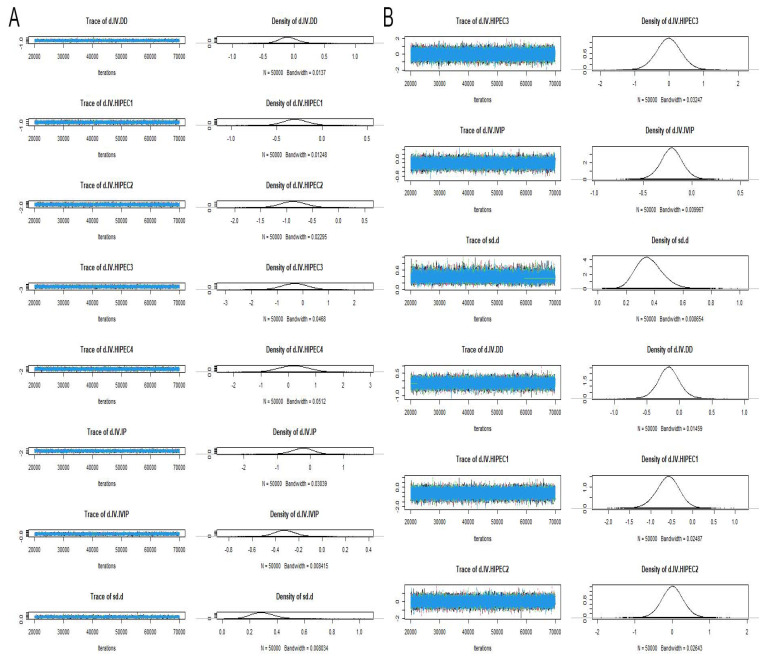
Trac and marginal density plots of OS (**A**) and PFS (**B**).

**Figure 4:**
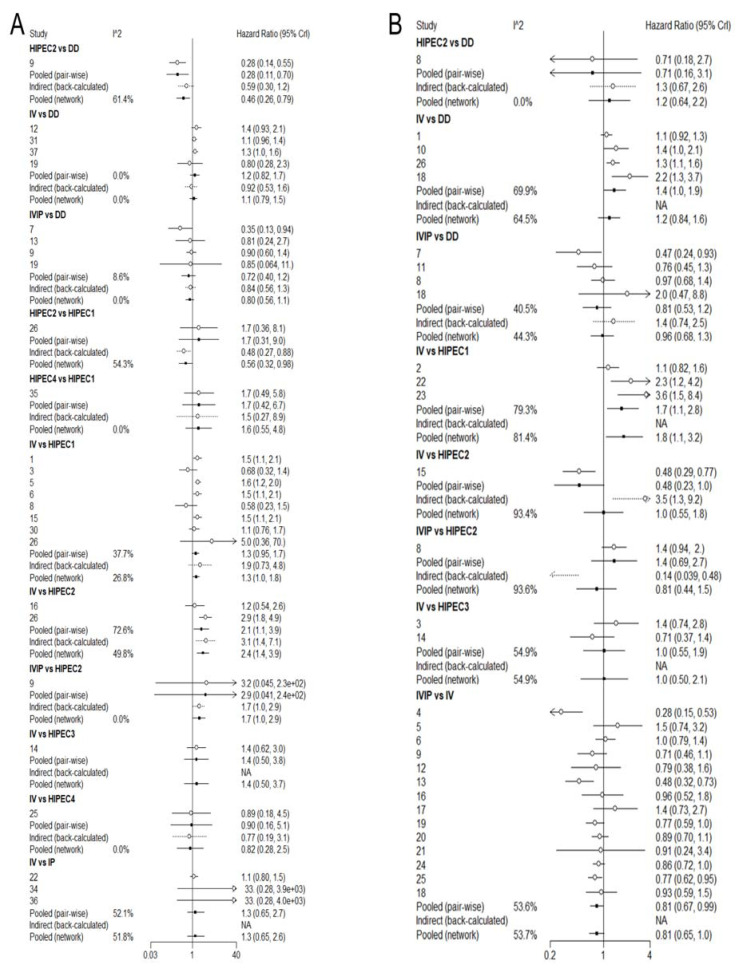
Forest plot of OS (**A**) and PFS (**B**). NA: not available.

**Figure 5:**
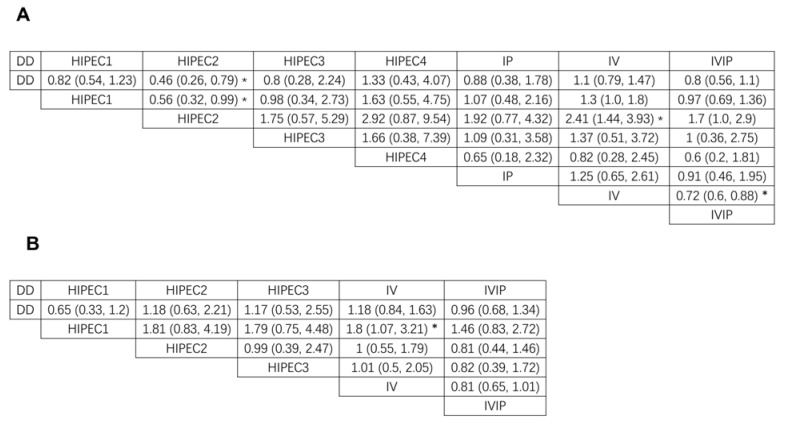
Network meta-analysis of OS (**A**) and PFS (**B**). *: *p* < 0.05.

Overall survival was reported in 31 studies with different chemotherapy regimens. Brooks–Gelman–Rubin, trace, and marginal density plots showed that the network meta-analyses converged on a solution within the 50,000 iterations after the burn-in period (Figure 3A). There was probably evidence of a difference favouring the group who received HIPEC2 and IVIP compared with IV (HR: 0.42, 95% CI 0.25 to 0.69, HR: 0.72, 95% CI 0.60 to 0.88, respectively). Our statistical results also showed evidence of a difference in favour of the group who received HIPEC2 compared with weekly dose-dense chemotherapy (HR: 0.46, 95% CI 0.26 to 0.79) and HIPEC1 (HR: 0.56, 95% CI 0.32 to 0.99), respectively. There was no evidence of differences between the other comparison groups. The results are shown in Figure 4A and Figure 5A. Based on NMA-derived ranking quantifying, HIPEC2 (76.17%) had the highest probability in improving OS, followed by HIPEC3 (14.23%), IP (5.22%), HIPEC4 (3.21%), HIPEC1(0.65%), IVIP (0.48%), DD (0.03%) and IV (0%).

According to SUCRA, HIPEC2 (76.17%) had the highest probability in improving OS, followed by acupoint catgut embedding (30.8%), and HRT (25.3%).

**Figure A1:**
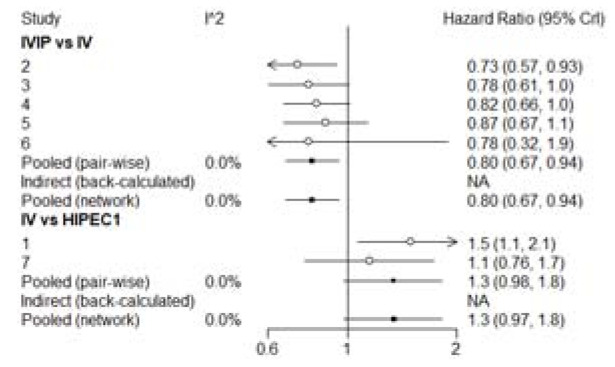
Forest plot of OS (RCTs).

**Figure A2:**
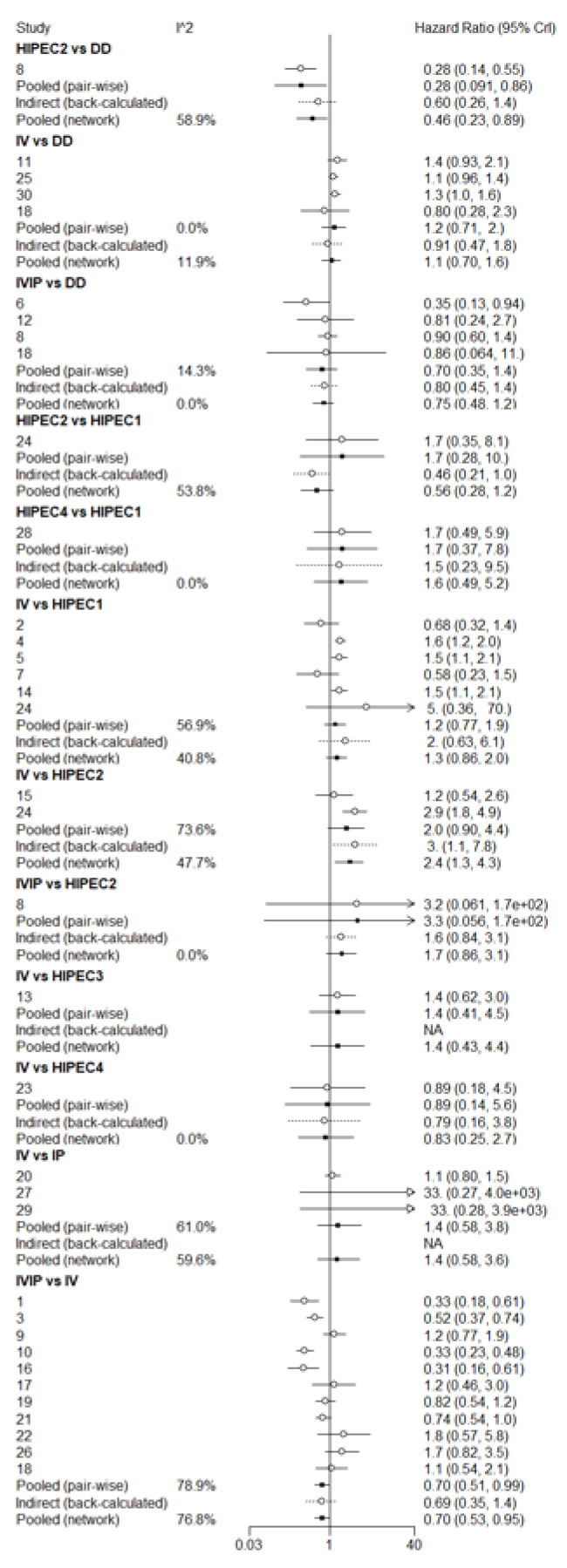
Forest plot of OS (non-RCTs).

Considering the combining RCTs with non-RCTs may introduce more bias. We performed a statistical analysis separately for RCTs and non-RCTs. The statistical results of the RCTs showed that IVIP compared with IV (HR: 0.80, 95% CI 0.67 to 0.94) may improve the OS (Figure A1). Statistical analyses of non- RCTs also yielded consistent conclusions, IVIP compared with IV (HR: 0.70, 95% CI 0.53 to 0.95). The statistical results of the non-RCTs also showed that HIPEC2 compared with DD and IV (HR: 0.46, 95% CI 0.23 to 0.89, HR: 0.42, 95% CI 0.23 to 0.77) may improve the OS, which was consistent with the results of the comprehensive analysis. The results are presented in Figure A2.

### 3.4. Progression-Free Survival (PFS)

Twenty-one studies have reported PFS with different chemotherapy regimens. Brooks–Gelman–Rubin, trace, and marginal density plots showed that the network meta-analyses converged on a solution within the 50,000 iterations after the burn-in period (Figure 3B). There was probably evidence of a difference favouring the group who received HIPEC1 compared with IV (HR: 0.55, 95% CI 0.31 to 0.94). There was no evidence of differences between the other comparison groups. The results are shown in Figure 4B and Figure 5B. Based on NMA-derived ranking quantifying the highest likelihood of providing maximal PFS benefit, HIPEC1 ranked first (80.52%), followed by HIPEC3 (7.30%), HIPEC2 (3.97%), IVIP (4.14%), DD (4.06%), IV (0.0%).

**Figure A3:**
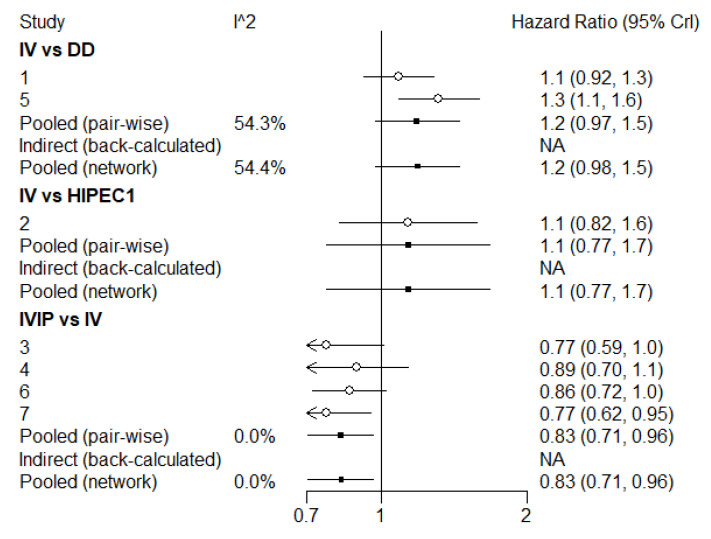
Forest plot of PFS (RCTs).

**Figure A4:**
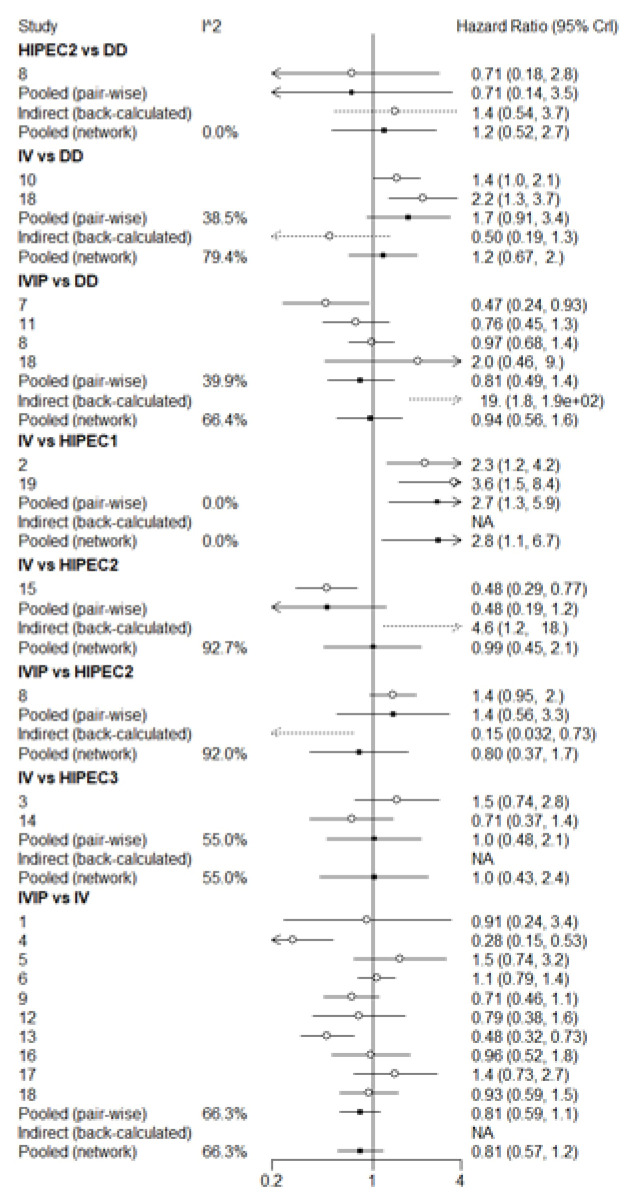
Forest plot of PFS (non-RCTs).

The statistical results of the RCTs showed that IVIP compared with IV (HR: 0.83, 95% CI 0.70 to 0.96) may improve the PFS (Figure A3). However, the statistical results for non-RCTs showed no difference. Moreover, the results of the non-RCTs showed that HIPEC1 may better benefit PFS compared with IV (HR: 0.36, 95% CI 0.15 to 0.91), which is consistent with the combined analysis results. The results are presented in Figure A4.

### 3.5. Adverse Events (AEs)

**Figure 6:**
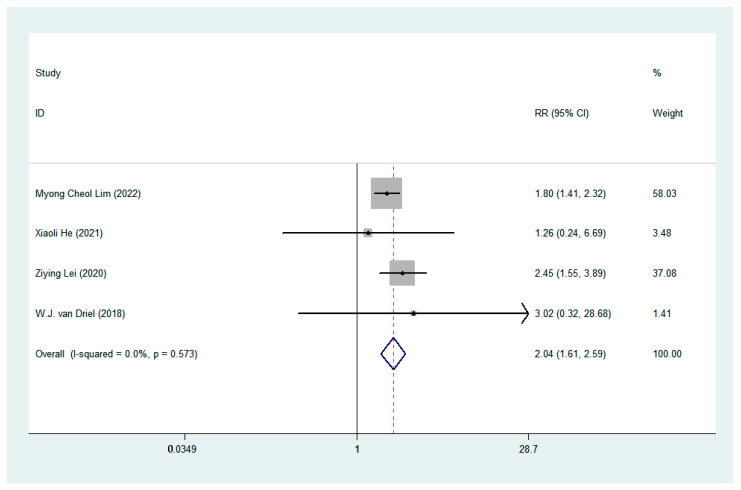
Forest plot of electrolyte disturbance.

**Figure 7:**
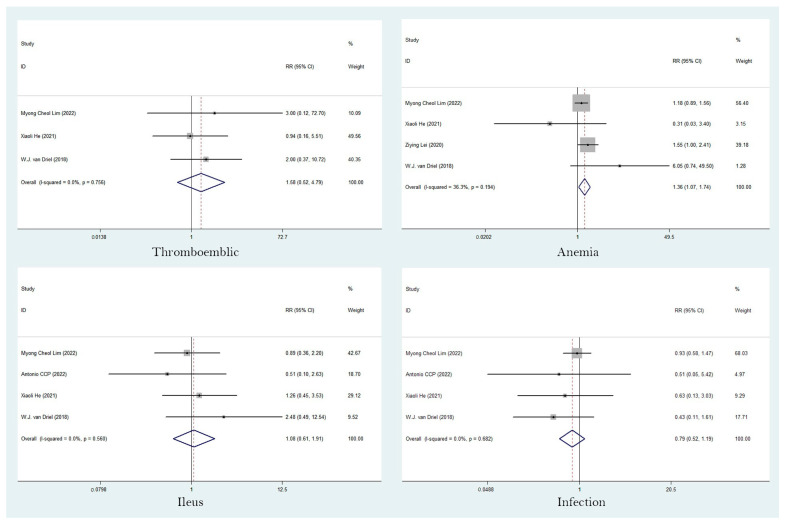
Forest plot of Adverse Events.

We compared AEs (≥grade 3) in HIPEC and non-HIPEC groups. A total of five AEs (include: anemia, electrolyte disturbance, ileus, thromboembolic and infection) were available for statistical analysis in including studies. Our statistical analysis results showed that compared with non-HIPEC groups, HIPEC had a higher risk of electrolyte disturbance (RR: 2.04, 95% CI 1.61–2.59, I^2^ = 0, *p* = 0.573, Figure 6). Heterogeneity was not presented across the studies. There was no significant difference in the other AEs between the two groups, and mild or no heterogeneity was present as shown in Figure 7.

### 3.6. Risk of Heterogeneity, Inconsistency and Bias

**Figure 8:**
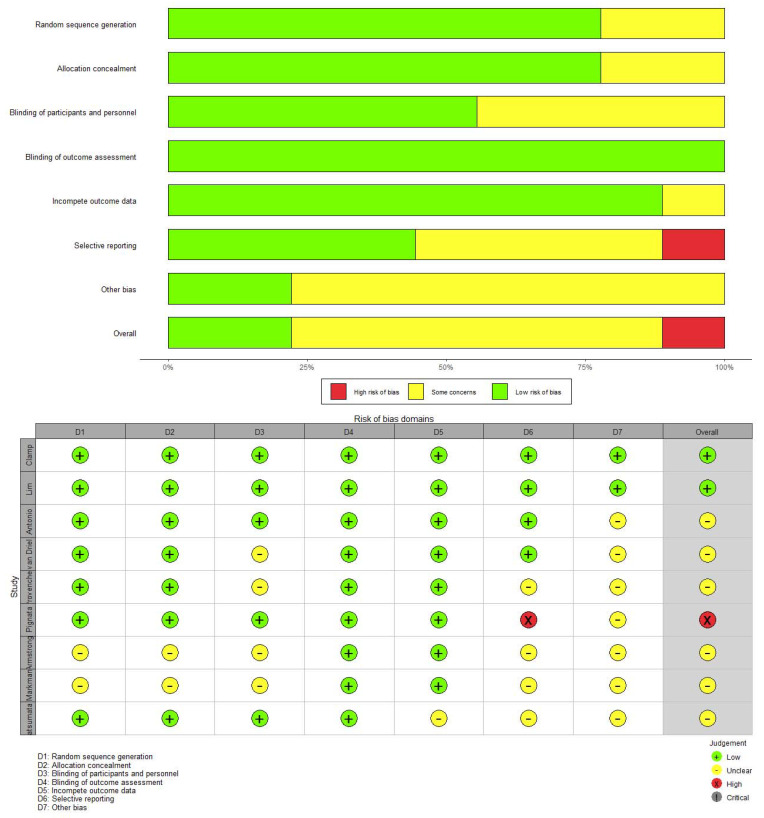
Risk of bias.

**Figure A5:**
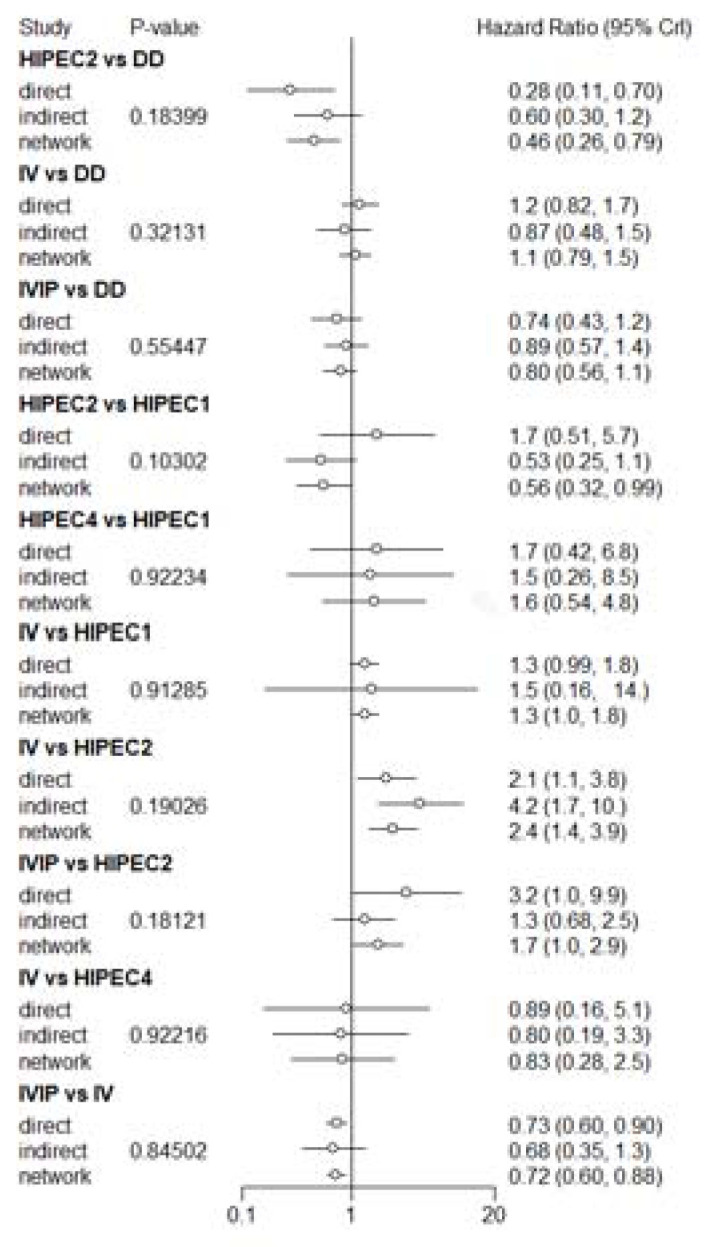
Result of node-splitting analysis for OS.

**Figure A6:**
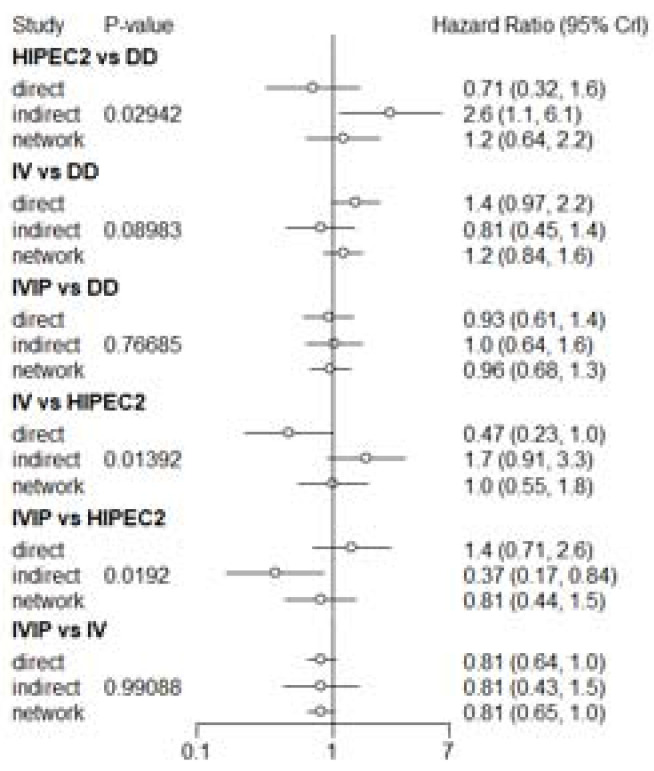
Result of node-splitting analysis for PFS.

Mild significant overall heterogeneity was observed in OS (Tau^2^ = 0.08, I^2^ = 21%) and PFS (Tau^2^ = 0.13, I^2^ = 14%) data sets. The node-splitting model of OS showed no local inconsistency in comparisons, all node splitting inconsistency *p* values were >0.05 (Figure A5). The node-splitting model of PFS showed local inconsistency between DD vs. HIPEC2, IV vs. HIPEC2 and IVIP vs. HIPEC2 (*p* < 0.05, Figure A6), but we observed no significant differences between the consistency model and the inconsistency model (DIC = 54.1, DIC = 52.8, respectively). The risk of bias in the included comparative studies was assessed using the NOS scale and the details of the scores are given in Table 2. The Cochrane Collaboration’s Risk of Bias Tool was used for the RCTs. The results are shown in Figure 8.

## 4. Discussion

Ovarian cancer is a disease that generally responds well to chemotherapy. However, newly diagnosed ovarian cancer is most commonly presented with a disease that is already at an advanced stage with a high recurrence rate and a low five-year survival rate. In recent years, many clinical studies have confirmed that targeted drugs such as poly (ADP-ribose) polymerase inhibitor (PARPi) can improve the prognosis of ovarian cancer patients. Platinum-based chemotherapy remains the first-line chemotherapy for ovarian cancer. There are various administration schedules of chemotherapy that have included IV, IP, HIPEC, and dose-dense regimens, etc.

This network meta-analysis compared survival outcomes from 32 studies involving 6347 patients. The results of the statistical analysis showed that HIPEC2 demonstrated a statistically significant OS benefit compared with IV, weekly dose-dense chemotherapy and HIPEC1. Compared with IV, IVIP was associated with a statistically significantly better likelihood of OS. Only three studies on HIPEC2 were included in our analysis, and only one study directly compared IV and DD individually. Thus, this statistical result is somewhat limited. Considering the combination of RCTs and non-RCTs may introduce more bias. We performed statistical analyses separately for RCTs and non-RCTs. Fortunately, the separate and combined analyses reach the same conclusion, suggesting that our statistical results have certain credibility. The IVIP chemotherapeutic regimen became the standard of care in North America when GOG 172 was published, which found a 16-month OS benefit for women receiving IVIP compared with those receiving IV only. Our statistical results are consistent with these RCTs [8]. For PFS, our statistical results only suggest a better PFS in ovarian cancer patients treated with HIPEC1 compared with DD. No evidence of any difference was observed between the other comparison groups.

Adverse events should also be of concern. The results of our analysis suggest that HIPEC may lead to a higher incidence of electrolyte disturbances. The review by S.P. Somashekhar et al. also suggested that electrolyte disturbances is one of the most common complications of HIPEC, with cisplatin having the highest rate of electrolyte disturbances compared with other drugs [46]. Cisplatin was the most common agent used in primary ovarian cancer.

On the other hand, the quality of life of patients after HIPEC also deserves our attention. Due to the limited articles available for statistical analysis, we did not analyze the quality of life in our network meta-analysis. One RCT we included holds the idea that no difference was observed in quality of life between patients with or without HIPEC [16]. The study by Kim JH et al. also reached a consistent conclusion [47].

In recent years, HIPEC has received growing attention in the treatment of primary advanced ovarian cancer. In a Dutch phase III trial, patients with FIGO stage III EOC who underwent HIPEC1 exhibited an improved overall survival (46 vs. 34 months) when compared with the standard treatment arm with comparable complication rates. However, there are other points of view as well. A small number of RCTs [16] showed no significant improvement in OS and PFS in stage III/IV EOC patients who with HIPEC1 compared with those at stage IV, and the other RCTs [15] showed that HIPEC did not improve PFS and OS in patients with advanced EOC. However, the subgroup analysis results give the idea that addition of HIPEC to interval cytoreductive surgery provided an improvement of OFS and OS. In 2013, a multicenter RCT conducted in Japan (JGOG 3016) concluded that dose-dense treatment (carboplatin AUC 6 mg/mL per min on day 1 and paclitaxel 80 mg/m² on days 1, 8, and 15) improves the prognosis of patients with stage II–IV ovarian cancer [41]. Other RCTs (ICON8) conducted in Europe hold the idea that weekly dose-dense chemotherapy did not improve overall or progression-free survival compared with standard 3-weekly chemotherapy [14]. Future studies will need to tailor patient selection, timing, and optimal regimens of chemotherapy regimens to improve the effectiveness of this specialized treatment in advanced ovarian cancer.

This network meta-analysis has potential limitations: (1) The literature included both comparative studies and RCTs, and there was relative heterogeneity among the studies. (2) We did not include studies of recurrent ovarian cancer. Moreover, our analysis only included platinum-based chemotherapy studies, and the studies which added other drugs, such as bevacizumab, were not included in the analysis. (3) Because the data cannot be split, we did not perform a subgroup analysis of the timing of chemotherapy regimens use, especially for HIPEC, which may have an important impact on the prognosis of ovarian cancer patients, which needs more clinical research in the future.

## 5. Conclusions

In summary, our statistical analysis suggests that the groups receiving HIPEC2 and IVIP had a better OS than the groups receiving IV. In addition, HIPEC2 may had a better OS compared with weekly dose-dense chemotherapy and HIPEC1. For PFS, our analysis only showed evidence of a difference favouring the group who received HIPEC1 compared with IV. HIPEC may lead to a higher incidence of electrolyte disturbances (≥grade 3). HIPEC therapy for advanced ovarian cancer is currently controversial.

## Figures and Tables

**Table 1:** Characteristics of included studies.

Study			Study Year	Stage	Study Design	Group	Number	Age, Years	OS HR (95% CI)	PFS HR (95% CI)	Follow-Up (Months)
1	2022	Clamp AR	2011–2014	IC–IV EOC	RCT	IV	522	63 (55–68)	0.87(0.73–1.05)	0.92 (0.78–1.09)	84
		[14]				DD	523	61 (54–67)			
2	2022	Lim MC	2010–2016	III/IV EOC	RCT	HIPEC1	92	52.0 (46–59.5)	0.87 (0.58–1.32)	0.88 (0.63–1.21)	69.4
		[15]				IV	92	53.5 (47.5–61)			
3	2022	Antonio CCP	2018–2020	III/IV EOC	RCT	HIPEC1	35	56 (29–75)	0.78 (0.32–1.88)	NA	60
		[16]				IV	36	65.5 (40–75)			
4	2021	He X	2012–2017	III EOC	retrospective study	HIPEC1	121	58.38	0.52 (0.35–0.78)	NA	90
		[17]				IV	76	55.2			
5	2021	Gruner M	2017–2020	III/IV HGSOC	cohort study	HIPEC3	21	63.1 ± 9.2	NA	0.69 (0.36, 1.38)	36
		[18]				IV	40	66.3 ± 9.5			
6	2021	Manning-Geist BL	2010–2014	IIIC EOC	retrospective study	IVIP (miliary)	41		0.33 (0.18–0.61)	0.28 (0.15–0.53)	>80
		[19]				IV (miliary)	49				
						IVIP (nonmiliary)	23		1.47 (0.7–3.09)	1.53 (0.74–3.19)	
						IV (nonmiliary)	55				
7	2021	Kim SR	2001–2015	IIIC/IV HGSOC	retrospective study	IVIP	91	55.1 ± 10.02	0.52 (0,36–0.73)	1.05 (0,79–1.4)	>120
		[20]				IV	180	59.8 ± 11.3			
8	2020	Lei Z	2010–2017	III EOC	retrospective study	HIPEC1	425	55.1	0.64(0.50–0.82)	NA	60
		[21]				IV	159	54.6			
9	2020	Ting WH	2006–2019	II–IV OC	retrospective study	IVIP	22	52.6 ± 7.4	0.35 (0.13–0.93)	0.47 (0.24–0.94)	>50
		[22]				DD	28	54.2 ± 8.7			
10	2020	Shibutani T	2006–2015	advanced EOC	retrospective study	DD	101	61 (35–79)	0.72(0.48–1.06)	0.69 (0.46–0.96)	>80
		[23]				IV	70				
11	2020	Murphy M	2010–2018	III OC	retrospective study	IVIP	44	63 (35–81)	0.81 (0.24–2.75)	0.76 (0.45–1.27)	>80
		[24]				DD	38	63 (35–87)			
12	2019	Rettenmaier M	2008–2015	AOC	retrospective study	HIPEC2	64	59.9	0.72(0.33–1.56)	0.71 (0.44–1.14)	>80
		[25]				IVIP	81	57.8	1.11(0.74–1.66)	0.97 (0.69–1.36)	
						DD	100	62.9			
13	2019	K. Bixel	2004–2017	III–IV OC	retrospective study	IVIP	37	59.7 (40–81)	1.22 (0.77–1.92)	0.71 (0.46–1.10)	60
		[26]				IV	97	66.3 (21–87)			
14	2018	Lee J	2006–2015	III/IV EOC	retrospective study	IDS + IVIP	42	60.0 (35–76)	0.61 (0.17–2.22	0.79 (0.38–1.64)	>60
		[27]				IDS + IV	24	59.0 (46–86)			
						PDS + IVIP	93	54.0 (25–81)	0.43(0.25–0.72)	0.48 (0.32–0.74)	
						PDS + IV	56	61.0 (37–84)			
15	2018	Ceresoli M	2010–2016	advanced EOC	retrospective study	HIPEC3	28	58.99	0.73 (0.33–1.60)	1.41 (0.73–2.73)	80
		[28]				IV	49	63.48			
16	2018	van Driel WJ	2007–2016	III EOC	RCT	HIPEC1	123	63 (56–66)	0.67(0.48–0.94)	NA	56.4
		[29]				IV	122	61 (55–66)			
17	2017	Mendivil AA	2008–2015	AOC	retrospective study	HIPEC2	69	59.8	0.84(0.38–1.84)	2.10 (1.29–3.42)	>50
		[30]				IV	69	62.9			
18	2017	Miller EM	2005–2016	III/IV OC	retrospective study	IVIP	49	56 ± 12	0.31 (0.16–0.62)	NA	120
		[31]				IV	54	58 ± 12			
19	2017	Eoh KJ	2006–2008	III/IV EOC	retrospective study	CIS-IVIP	21	53 (37–72)	1.18 (0.46–3.04)	0.96 (0.52–1.79)	120
		[32]				CAR-IVIP	16	52 (41–65)	1.7 (0.82–3.54)	1.41 (0.73–2.73)	
						IV	121	58 (22–82)			
20	2017	Provencher DM		IIB–IVA EOC	RCT	CIS-IVIP	102	62 (40–82)	0.73 (0.57–0.93)	0.77 (0.59–1.02)	50
		[33]				CAR-IVIP	61	64.9 ± 10.5	0.78 (0.61–1.01)	0.89 (0.69–1.13)	
						IV	72	61 (29–78)			
21	2017	Sioulas VD	2001–2010	III EOC	retrospective study	IVIP	61	64.9 ± 10.5	0.82 (0.54–1.25)	NA	53
		[34]				IV	120	57.9 ± 9.8			
22	2017	Nicoletto MO	2006–2015	AOC	retrospective study	IP	33	62.6	0.90 (0.65–1.24)	NA	40
		[35]				IV	66	66.7			
23	2016	Mueller JJ	2008–2013	III–IV OC	retrospective study	IVIP	48	60 (34–76)	1.07 (0.55–2.09)	0.93 (0.6–1.45)	>60
		[36]				DD	17	64 (36–80)	1.25 (0.47–3.37)	1.92 (1.0–3.96)	
						IV	63	66 (38–86)			
24	2016	Cascales-Campos P	2008–2015	III/IV EOC	retrospective study	HIPEC4	60	59.43 ± 1.4	0.59(0.17–2.03)	NA	>80
		[37]				HIPEC1	51	60.53 ± 1.5			
25	2014	Al Mutairi NJ	2007–2009	III/IV EOC	retrospective study	IVIP	16		0.74 (0.03–17.44)	0.91 (0.24–3.44)	26.2
		[38]				IV	18				
26	2014	Pignata S	2008–2012	IC–IV EOC	RCT	IV	522	63 (55–68)	0.87 (0.73–1.05)	0.92 (0.78–1.09)	69
		[39]				DD	523	61 (54–67)			
27	2014	Yoon JY	2003–2012	III EOC	retrospective study	IP	37		0.03 (0.01–145.55)	NA	60
		[40]				IV	26				
28	2013	Katsumata N	2003–2005	II–IV OC	RCT	DD	312		0.79 (0.63–0.99)	0.76 (0.62–0.91)	76.8
		[41]				IV	319				
27	2010	Kim SW	2006–2007	OC	retrospective study	IVIP	19	54 ± 14	1.82 (0.57–5.77)	NA	50
		[42]				IV	34	52 ± 13			
29	2009	Francisco C	1997–2004	III EOC	retrospective study	HIPEC4	14	54 (28–68)	1.12 (0.23–5.82)	NA	>60
		[43]				IV	12	54 (30–67)			
30	2007	Bae JH	1995–2004	IC-IIIC EOC	retrospective study	HIPEC2	45	50.1 ± 12.4	0.34 (0.24–0.66)	0.44 (0.24–0.81)	>120
		[44]				HIPEC4	22	49.5 ± 8.6	0.20 (0.05–0.82)	0.28 (0.12–0.67)	
						IV	29	50.0 ± 11.7			
31	2006	Armstrong DK	1998–2001	III EOC	RCT	IV	210		0.75 (0.58–0.97)	0.80 (0.64–1.00)	60
		[8]				IVIP	205				
32	2001	Markman M	1992–1995	III OC	RCT	IVIP	235		0.87 (0.67–1.14)	0.77 (0.62–0.95)	60
		[45]				IV	227				

Note: EOC: Epithelial ovarian cancer; OC: Ovarian cancer; HGSOC: High-grade serous ovarian cancer; AOC: Advanced ovarian cancer; OS: Overall survival; PFS: Progression-free survival; RCT: Randomized controlled trials; HIPEC: Hyperthermic intraperitoneal chemotherapy; IV: Intravenous chemotherapy; IP:Intraperitoneal chemotherapy; IVIP: Intravenous plus intraperitoneal chemotherapy; DD: weekly dose-dense chemotherapy; NA: not available.

**Table 2:** The NOS score of the included literature.

Study	Year	Selection				Comparability	Assessment of Outcome	Follow-Up	Adequacy of Follow-Up	Scores
		1	2	3	4					
**e X**	2021	*	*	*	*	*	*	*	*	8
**Gruner M**	2021	*	*	*	*	*	*		*	7
**Manning-Geist BL**	2021	*	*	*	*	*	*	*	*	8
**Kim SR**	2021	*	*	*	*	*	*		*	7
**Lei Z**	2020	*	*	*	*	*	*	*	*	8
**Ting WH**	2020	*	*	*	*	*	*	*	*	8
**Shibutani T**	2020	*	*	*	*	*	*	*	*	8
**Murphy M**	2020	*	*	*	*	*	*	*	*	8
**Mark A**	2019	*	*	*	*	*	*	*	*	8
**K. Bixel**	2019	*	*	*	*	*	*		*	7
**Lee J**	2018	*	*	*	*	*	*		*	7
**Ceresoli M**	2018	*	*	*	*	*	*		*	7
**Mendivil AA**	2017	*	*	*	*	*	*	*	*	8
**Miller EM**	2017	*	*	*	*	*	*	*	*	8
**Eoh KJ**	2017	*	*	*	*	*	*	*	*	8
**Mueller JJ**	2017	*	*	*	*	*	*	*	*	8
**Sioulas VD**	2017	*	*	*	*	*	*	*	*	8
**Nicoletto MO**	2017	*	*	*	*	*	*	*	*	8
**Cascales-Campos P**	2016	*	*	*	*	*	*	*	*	8
**Yoon JY**	2014	*	*	*	*	*	*	*	*	8
**Al Mutairi NJ**	2014	*	*	*	*	*	*	*	*	8
**Kim SW**	2010	*	*	*	*	*	*		*	7
**Francisco C**	2009	*	*	*	*	*	*	*	*	8
**Bae JH**	2007	*	*	*	*	*	*	*	*	8

## Data Availability

Not applicable.

## Supplementary Materials

The following supporting information can be downloaded at: https://www.mdpi.com/article/10.3390/jcm12031111/s1, File S1. Detailed search strategies.
