# Supplementary material for: Comparative Survival Outcomes of Hyperthermic Intraperitoneal Chemotherapy, Intraperitoneal Chemotherapy and Intravenous Chemotherapy for Primary Advanced Ovarian Cancer: A Network Meta-Analysis
Source: J Clin Med. 2023 Jan 31;12(3):1111. doi: 10.3390/jcm12031111 (PMC9917421; doi:10.3390/jcm12031111)
Supplement: Supplementary file 1 [file jcm-12-01111-s001.zip › File S1. Detailed search strategies.pdf]

PUBMED search strategy

#1 ("Ovarian Neoplasms"[Mesh]) OR (("Neoplasms"[Mesh] OR carcinoma*[tw] OR neoplas*[tw] OR tumour*[tw] OR sarcoma*[tw] OR adenoma*[tw] OR tumor*[tw] OR cancer*[tw] OR oncolog*[tw] OR malignan*[tw] OR metasta*[tw] OR carcinogen*[tw] OR oncogen*[tw]) AND (ovarian*[tw] OR ovary[tw]))

#2 (chemotherapy[tw] OR chemoperfusion[tw]) AND (intraperitoneal*[tw] OR intra-peritoneal*[tw] OR peritoneal*[tw])

#3 HIPEC[tw]

#4 #2 OR #3

#5 #1 AND #4

Web of Science search strategy

#1 TS=(Ovarian Neoplasms OR Neoplasms OR carcinoma*OR neoplas* OR tumour* OR sarcoma* OR adenoma* OR tumor*OR cancer* OR oncolog* OR malignan* OR metasta* OR carcinogen* OR oncogen*) AND TS=(ovarian* OR ovary)

#2 TS=(chemotherapy OR chemoperfusion) AND TS=(intraperitoneal* OR intra-peritoneal* OR peritoneal*)

#3 TS=(HIPEC)

#4 #2 OR #3

#5 #1 AND #4

Scopus search strategy

(((TITLE-ABS-KEY(intraperitoneal* OR intra-peritoneal* OR peritoneal*)) AND (TITLE-ABS-KEY(chemotherapy OR chemoperfusion))) OR (TITLE-ABS-KEY(HIPEC))) AND ((TITLE-ABS-KEY(Neoplasms OR carcinoma*OR neoplas* OR tumour* OR sarcoma* OR adenoma* OR tumor*OR cancer* OR oncolog* OR malignan* OR metasta* OR carcinogen* OR oncogen*))AND (TITLE-ABS-KEY (ovarian* OR ovary)))

CENTRAL (Cochrane Central Registry of Controlled Trials)

#1 MeSH descriptor: [Ovarian Neoplasms] explode all trees

#2 (Neoplasms OR carcinoma*OR neoplas* OR tumour* OR sarcoma* OR adenoma* OR tumor*OR cancer* OR oncolog* OR malignan* OR metasta* OR carcinogen* OR oncogen*):ab

#3 (ovarian* OR ovary):ab

#4 #2 AND #3

#5 #1 OR #4

#6 (chemotherapy OR chemoperfusion OR HIPEC) :ab

#7 (intraperitoneal* OR intra-peritoneal* OR peritoneal*):ab

#8 #6 AND #7

#9 #5 AND #8

Embase

#1 (neoplasms:ab OR carcinoma*:ab OR neoplas*:ab OR tumour*:ab OR sarcoma*:ab OR adenoma*:ab OR tumor*:ab OR cancer*:ab OR oncolog*:ab OR malignan*:ab OR metasta*:ab OR carcinogen*:ab OR oncogen*:ab) AND (ovarian*:ab OR ovary:ab)

#2 (chemotherapy:ab OR chemoperfusion:ab) AND (intraperitoneal*:ab OR 'intra peritoneal*':ab OR peritoneal*:ab)

#3 HIPEC:ab

#4 #2 OR #3

#5 #1 AND #4
